# Self-reported validity of self-presentation on social media sites and its association with affective disorder

**DOI:** 10.1192/j.eurpsy.2021.913

**Published:** 2021-08-13

**Authors:** K. Rydahl, M. Speed, S. Østergaard

**Affiliations:** Department Of Affective Disorders, Aarhus University Hospital, Aarhus N, Denmark

**Keywords:** unipolar depression, Cognitive bias, Self-Presentation, social media

## Abstract

**Introduction:**

Individuals with affective disorders, who are prone to negative cognitive bias, may be particular vulnerable to positively biased presentations by other social media users. The degree of positive bias in self-presentations on social media sites is however poorly understood

**Objectives:**

To investigate the validity of self-presentation on social media sites and its association with affective disorder

**Methods:**

Individuals aged 18-75 receiving treatment at the outpatient clinic for affective disorders at Aarhus Hospital or at two general practices were invited to participate in a survey focusing on social media use. Two core questions were: “To what extent do your social media content reflect your real life” and ”To what extent do others’ social media content reflect their real lives”. Response was provided on a likert scale with the following steps: “much more negative” (1), “more negative” (2), “the same as” (3), “more positive” (4) and “much more positive” (5) than real life. Based on these responses on bias, we calculated a bias ratio (validity of own self-presentation/validity of others’ self-presentation). The association between unipolar depression, bipolar disorder and bias ratio>1 was investigated using logistic regression with adjustment for age and sex

**Results:**

A total of 183 individuals with unipolar depression, 119 with bipolar disorder and 186 controls participated in the study. Unipolar depression was associated with a bias ratio >1 (OR: 3.4, 95%CI: 1.2;9.9)

**Conclusions:**

Individuals with unipolar depression are prone to consider their self-presentation as more positively biased compared to others’ self-presentation. This may shape the impact of social media use on these individuals.

